# A Philosophical Perspective on the Relation between Cortical Midline Structures and the Self

**DOI:** 10.3389/fnhum.2013.00536

**Published:** 2013-09-02

**Authors:** Kristina Musholt

**Affiliations:** ^1^Department of Philosophy, Logic and Scientific Method, London School of Economics and Political Science, London, UK

**Keywords:** self-consciousness, consciousness, cortical midline structures, phenomenology, schizophrenia, personal level, subpersonal level

## Abstract

In recent years there has been increasing evidence that an area in the brain called the cortical midline structures (CMSs) is implicated in what has been termed self-related processing. This article will discuss recent evidence for the relation between CMS and self-consciousness in light of several important philosophical distinctions. First, we should distinguish between being a self (i.e., being a subject of conscious experience) and being aware of being a self (i.e., being able to think about oneself as such). While the former consists in having a first-person perspective on the world, the latter requires the ability to explicitly represent one’s own perspective as such. Further, we should distinguish between being aware of oneself “as subject” and being aware of oneself “as object.” The focus of existing studies investigating the relation between CMS and self has been predominantly on the ability to think about oneself (and in particular thinking of oneself “as object”), while the more basic aspects involved in being a self have been neglected. However, it is important to widen the scope of the cognitive neuroscience to include the latter, not least because this might have important implications for a better understanding of disorders of the self, such as those involved in schizophrenia. In order to do so, cognitive neuroscience should work together with philosophy, including phenomenology. Second, we need to distinguish between personal and subpersonal level explanations. It will be argued that although it is important to respect this distinction, in principle, some subpersonal facts can enter into constitutive conditions of personal-level phenomena. However, in order for this to be possible, one needs both careful conceptual analysis and knowledge about relevant cognitive mechanisms.

## Introduction

Self-consciousness is a topic that is one of the classical concerns of philosophy. More recently, it has also begun to take center stage in cognitive science studies, including neuroimaging studies. In particular, in recent years there has been increasing evidence that an area in the brain called the cortical midline structures (CMSs) is implicated in what has been termed self-related processing. This article will discuss recent evidence for the relation between CMS and self-consciousness in light of several important philosophical distinctions.

After briefly summarizing what is known to-date about the relation between the self and CMS and raising some general concerns regarding the attempt to localize the self in the brain, it will first be argued that we should distinguish between *being a self* (i.e., being a subject of conscious experience) and *being aware of being a self* (i.e., being able to think about oneself). While the former consists in having a first-person perspective on the world, the latter requires the ability to explicitly represent one’s own perspective as such. This, in turn, requires an awareness of other minds, and the ability to contrast one’s own perspective with that of others. It will be argued that the focus of existing studies investigating the relation between CMS and self has been predominantly on the ability to think about oneself, while the more basic aspects involved in being a self have been neglected. It will be argued further that it is important to widen the scope of cognitive neuroscience to include the latter, not least because this might have important implications for a better understanding of disorders of the self, such as those involved in schizophrenia. In order to do so, cognitive neuroscience should work together with philosophy, including phenomenology. It will also be argued that we should distinguish between thinking of oneself “as subject” and thinking of oneself “as object” and that it might be interesting to target this distinction in future empirical studies.

Second, the article will ask what and how exactly (if anything) the study of CMS can teach us about self-consciousness. It will be argued that we need to distinguish between personal and subpersonal level explanations. At the personal level we refer to a person’s conscious experience and mental states in order to make intelligible the behavior of a person by providing the reasons the person might have for acting in the way that they do. Subpersonal level explanations on the other hand provide information about the physiological or computational enabling conditions of personal-level phenomena. Properties at the subpersonal level are neither conscious, nor do they make reference to mental states or reasons. This raises the question as to how we should think of the relation between these two levels of explanation. Can neuroscience only ever provide us with correlations or causal relations between personal and subpersonal level phenomena? Or can a brain state, such as a particular pattern of activation in CMS, be part of the constitutive basis of being in a psychological state, such as a state of self-awareness? It will be argued that, in principle, some subpersonal facts can enter into constitutive conditions of personal-level phenomena. However, in order for this to be possible, one needs both careful conceptual analysis and knowledge about relevant cognitive mechanisms (such as neurocomputational mechanisms).

## The Self and Cortical Midline Structures

It seems that in recent years social cognitive neuroscience has made much progress in identifying the neural correlates of self-awareness. In particular, functional neuroimaging (fMRI) studies have demonstrated the involvement of CMS, including the ventral medial prefrontal cortex (vMPFC), dorsal MPFC (dMPFC), and parietal/posterior cingulate cortex (PCC), as well as the anterior CMS (e.g., ACC) in self-related processing (e.g., Gusnard et al., 2001; Kelley et al., 2002; Northoff and Bermpohl, 2004; Platek et al., 2004; Ochsner et al., 2005; Northoff et al., 2006; Yaoi et al., 2009).

The involvement of CMS in the processing of self-related stimuli seems to be independent of the sensory domain in which the self-related stimulus is being represented (e.g., auditory, visual, mental), and of the task domain (e.g., verbal, spatial, memory, emotional, facial, social, agency) used in any specific study. More recently, a study by Moran et al. (2009) also found that CMS are involved both in the explicit and implicit processing of self-related stimuli, that is, in situations that involve self-relevant stimuli, regardless as to whether subjects were asked explicitly to engage in self-referencing.

Interestingly, the brain areas associated with the processing of self-related stimuli seem to overlap with what has been called the “default-mode network” of the brain. This network is thought to be involved in the processing of self-generated stimuli (as opposed to stimuli from the external world) and is thought by some to instantiate “the self” (Gusnard et al., 2001; Wicker et al., 2003; Schneider et al., 2008)^1^.

However, here, I want to raise some doubts regarding the possibility of locating the self in the brain.

For one thing, it has been pointed out that the relevant studies often do not adequately distinguish familiarity (i.e., the personal familiarity with a place, person, or other stimulus, which may elicit autobiographical memories or emotional reactions) from self-relatedness (Gillihan and Farah, 2005), and between self-specific and non-self-specific task demands (such as the recruitment of attention or executive control functions, which might be required in order to make evaluative or recognitional judgments) (Legrand and Ruby, 2009). Specifically, Legrand and Ruby, 2009, p. 270) argue that self-related tasks, which involve self-evaluative judgments, are not self-specific, because “the evaluative processes enabling identification, attribution, and reflection upon a subject are not different for self and others.”

These concerns were partly addressed in a recent meta-analysis by Qin and Northoff, 2011, p. 1223). The aim of this study was to investigate “the relationship between brain activity related to the processing of self-specific, personally familiar, and other (non-self and non-familiar) stimuli,” while controlling for task- and stimulus-dependent effects. The study demonstrated that there is indeed overlap between the processing of self-related, other-related, and familiar stimuli in several regions of the CMS (in particular in the MPFC and PCC). However, the perigenual anterior cingulate cortex (PACC) seems to be recruited specifically in the processing of self-related stimuli, as well as during resting-state conditions. The study also partly confirmed the hypothesis put forward by Legrand and Ruby (2009) that general task demands (such as those involved in evaluative and recognitional judgments) lead to an activation of CMS (in particular MPFC and PCC/precuneus), suggesting that such activation is not specific for the processing of self-related stimuli. Again, the PACC seems to be exempt from this, though Qin and Northoff (2011) point out that due to a lack of control in the original studies their results are not conclusive with respect to this point.

In sum, this meta-analysis suggests that the PACC seems to be recruited specifically for the processing of self-related stimuli as well as in resting-state activity, while other regions of the CMS, such as the MPFC and the PCC are involved in the evaluation of stimuli as well as in the processing of familiarity and the distinction between self and other.

Moreover, other areas of the brain were also identified as being involved in the processing of self-related stimuli. These include the lateral prefrontal cortex and the left anterior insula. These areas have also been suggested to be involved in self-referential processes in previous studies (Keenan et al., 2001; Platek et al., 2008; Modinos et al., 2009).

Taken together these results suggest some involvement of CMS (in particular the PACC) in the processing of self-related stimuli. However, the results also indicate the involvement of CMS in other processes, such as general (i.e., non-self-related) evaluative or recognitional judgments, as well as the involvement of other areas in self-related processing, thus raising doubts as to how specific the relation between CMS and self is. What this shows is that we have to be very careful with regard to claims suggesting that the self is “located” in particular areas of the brain.

Notice also that it is common in the literature on self and CMS to equate self-related with self-specific processing (accordingly, these two terms have been used somewhat interchangeably in this section). However, as mentioned above, according to Legrand and Ruby (2009), self-relatedness should be distinguished from self-specificity. That is to say that on their view one should distinguish between the process of judging a stimulus to be self-related and the self-specifying functional processes that implement a self/non-self distinction at a more fundamental level (and which provide the basis for judgments of self-relatedness) (also, see Christoff et al., 2011).

This distinction, which we will return to in the following, is related to a more general concern regarding the attempt to locate the self in the brain, namely the fact that the phenomenon of self-awareness is multi-faceted, involving, for example, the distinction between being a self (i.e., being the subject of conscious experience) and being aware of being a self (i.e., possessing the ability to think about oneself), as well as between synchronic and diachronic aspects of self-awareness (where the former indicates awareness of oneself at a given time and the latter awareness of oneself across time), or between cognitive, agentive, and affective elements of self-awareness. Given this multi-faceted nature, it is not surprising that recent results shed doubt on the notion of a particular location for “the self.” In fact, given the multi-faceted and complex nature of the self and self-awareness, we should expect both self-related and self-specific activity to be broadly distributed across the brain, involving diverse affective and cognitive processes. In the following section, I am going to take a closer look at the first distinction – that is, the distinction between being a self and being aware of being a self – with the aim of evaluating the implications of this distinction for studies of the relation between CMS and the self.

## Being a Self vs. Being Self-Aware

Within philosophy different notions of self and self-awareness can be distinguished. One of the crucial distinctions on my view is the distinction between being a self (i.e., being the subject of conscious experience) and being aware of being a self (i.e., being able to think about oneself).

The former consists in having a first-person perspective on the world, that is, a particular point of view. In contrast, the latter consists in the ability to think about oneself as such, that is, to explicitly represent one’s perspective as such. Thus, while the former can be characterized as being “world-directed,” the latter is “self-directed.” The ability to think about oneself, in turn, requires conceptual abilities, an awareness of other minds, and the ability to contrast one’s own perspective with that of others (Musholt, 2012).

It is important not to conflate these two notions with each other. For while it is reasonable to assume that every conscious experience is experience from a subjective point of view, this subjective point of view need not itself be part of the representational content of experience. It is one thing to have a perspective on the world (or to be aware of different ways in which one can interact with the world), but it is quite another to be aware of having this perspective (or to be aware of oneself as an agent) (Baker, 1998, 2012). In fact, it would put an unnecessary cognitive burden on the organism to always represent its own perspective on the world. Rather, precisely because an organism’s perception of the world is always from its own perspective, this fact itself can “drop out” of the content of conscious experience – the self can be thought of as an “unarticulated constituent” (Perry, 1998) of experience. That is to say that while perception contains implicitly self-related information (for instance, the objects in one’s environment are always presented in a certain distance and orientation from oneself), this does not mean that the self is part of the explicit representational content of experience (Musholt, 2013). The explicit representation of one’s own perspective only becomes important once an organism has an understanding of the perspective of others and wants to contrast its own perspective with that of others (Beckermann, 2003; Musholt, 2012). (This ability, in turn, can enable a host of other important abilities, such as the ability to deceive as well as the ability to cooperate by sharing intentions.) Accordingly, self-consciousness and intersubjectivity can be regarded as two sides of the same coin. Interestingly, some studies have found that some parts of the CMS, as well as other areas of the brain, such as the temporoparietal junction (TJP) are activated in the reflection on both one’s own and the mental states of others (e.g., Mitchell et al., 2005; Uddin et al., 2007; Lombardo et al., 2010).

That being said, the way we think about ourselves is not necessarily the same as the way we think about others. Rather, following a distinction introduced by Wittgenstein (1958), one can distinguish two-ways in which a subject can be self-aware. In one case, the subject thinks of herself “as subject.” This is the case, for example, when the subject experiences a mental state – such as a pain, for example – and on the basis of this experience ascribes the state to herself by using the first-person concept: “I have pain.” Although mental state concepts, such as the concept “pain,” can be applied both to self and other (reflecting a common conceptual schema), each of us also has privileged access to their own pain state. While I can ascribe pain to you by inferring from your behavior or your facial expression that you must be in pain, I do not need to rely on behavioral observation or inference in order to ascribe pain to myself. Rather, the access to my pain is direct, immediate, and non-inferential. However, the subject can also think of herself “as object,” that is, in the same way as she would think of another (i.e., by adopting the perspective of another onto herself). For example, when the subject observes herself in the mirror and ascribes to herself the property of being tall, she does this on the same basis as she would in the case of another – in this case by looking at herself. Self-ascriptions of the first kind have special significance for self-knowledge, because they are often taken to be “immune to error through misidentification relative to the first-person pronoun” (Shoemaker, 1968). This is to say that when ascribing properties to oneself by taking oneself “as a subject,” one might be wrong with regard to the property one is self-ascribing, but one cannot be wrong with regard to the subject of this self-ascription – oneself. The reason for this is because the relevant thought does not contain an identification component – I do not need to identify myself in order to know that I feel pain (Evans, 1982). In contrast, when I look at myself in the mirror and judge that I am tall, I am identifying the person that I see in the mirror with myself. Hence, this thought is liable to error through misidentification.

Note that these different types of self-representation are not distinguished in terms of their *content*, but rather in terms of their *mode* of presentation. When thinking of oneself “as subject” one adopts a first-person mode of presentation, that is to say, a mode that is specific to ways of gaining information about oneself (Perry, 2002; Recanati, 2012). In contrast, when thinking of oneself “as object” one adopts a third-person mode of presentation, that is to say a mode of presentation that is not specific to ways of gaining information about oneself. In the former case the resulting self-ascription will be immune to error through misidentification, as no identification is required, whereas in the latter case it will not. For example, I can ascribe a mental state, a certain belief state, say, to myself either on the basis of my awareness of the mental state in question (from the first-person perspective), or on the basis of adopting another’s perspective onto myself (from the third-person perspective), for instance by reflecting of my past behavior and concluding that I must have a certain belief without having been aware of it. The content of the self-ascription will be the same in both cases, namely “I believe x,” whereas the mode – the basis upon which the self-ascription is made – will be different. Similarly, I can judge that my legs are crossed either on the basis of proprioceptive experience (from the first-person perspective) or on the basis of looking at them in the mirror (from the third-person perspective). Again, the content of the judgment “My legs are crossed” will be the same in both cases, but the mode will be different. In the first case my judgment simply makes explicit the fact that proprioceptive information necessarily concerns the subject of experience – no self-identification is required. In the second case, the subject identifies the image in the mirror with herself.

Thus, we should distinguish perspectivalness and other aspects of subjectivity that constitute part of the structure of consciousness as such (and that provide the basis for the ability to think about oneself “as subject”), and specific types of conscious experience, namely experience or awareness of the self (where this is to be understood in terms of explicit self-representation, or thinking about oneself as such). Moreover, while the latter requires conceptual abilities and is closely tied to our ability to ascribe mental states to others, this is not say that the way in which we represent or think about ourselves is necessarily the same in which we represent our think about others. Rather, we can distinguish between thinking about oneself “as subject” (in the first-person mode) and thinking about oneself “as object” (in the third-person mode).

The distinction between “being a self” and “being aware of being a self” is related to the distinction between pre-reflective and reflective self-awareness (e.g., Zahavi, 2005; Legrand, 2007a; also, see Legrand, 2003, 2006). However, there are also important differences. For one thing, while the notion of possessing pre-reflective self-awareness is largely equivalent to the notion of “being a self” (i.e., being a subject of conscious experience), I prefer to reserve the term “self-awareness” for the ability to refer to oneself in thought, which, in turn, requires the ability to explicitly represent oneself. However, this issue can largely be regarded as a terminological matter for the purposes of this paper (for a detailed discussion, see Musholt, 2013). More importantly, though, the notion of reflective self-awareness is not quite the same as the notion of being aware of being a self (i.e., the ability to think about oneself), because while Legrand and others seem to think that reflective self-awareness (and thus explicit self-representation, or thinking about oneself) necessarily implies being aware of oneself “as object” (see in particular Legrand, 2007a), as I have argued above, one can think about oneself (and thus explicitly represent oneself) *either* “as subject” *or* as “object,” depending on whether the explicit self-representation is based on a first-person mode of presentation (i.e., a way of gaining information that is specific to the self), or on a third-person mode of presentation (i.e., a way of gaining information that is not specific to the self). Therefore, the three-way distinction between being a self on the hand and representing or thinking about oneself either “as subject” or “as object” on the other hand that I have introduced here does not map exactly onto the two-way distinction between pre-reflective and reflective self-awareness.

Now, which of these notions (if any) are in play in studies investigating the relation between CMS and the self?

Most of the relevant neuroimaging studies so far have relied on the explicit judgments of self-relatedness. They usually involve the presentation of personality traits which subjects have to rate as being more or less self-related on a given scale. [Though note that (Heinzel et al., 2006; Northoff et al., 2009; Qin and Northoff, 2011) use a broader notion of self-relatedness which includes knowledge of one’s own body, knowledge of psychological traits, and episodic memory, as well as more generally the relationship to specific stimuli in the environment, such as the degree of personal relevance and meaning attached to emotional pictures.] It seems clear that in these kinds of studies what is being probed is (explicit) self-consciousness. Participation in these studies requires subjects to have a concept of themselves and to be able to reflect on questions such as whether a certain personality trait belongs to them or not. Even knowledge of one’s bodily features, psychological traits, and memories requires the ability to think about oneself. Moreover, insofar as specific stimuli are rated as being more or less self-relevant or meaningful, they also need to be reflected upon and to be put in relation to oneself – which, again, requires thinking about oneself. Thus, it seems clear that by and large the studies that are investigating the relation between CMS and the self are investigating explicit self-awareness, and not the more basic aspect of the subjectivity of conscious experience, of being a self or a subject of experience (cf. Legrand, 2003, 2007a; Legrand and Ruby, 2009; Christoff et al., 2011.) While recently, studies have begun to address aspects of self-awareness that do not rely on explicit self-reflection (Moran et al., 2009), these studies still rely on explicit, conceptual knowledge about the self (such as knowledge about personal semantic information).

What seems less clear is whether subjects who engage in this task do so by thinking of themselves as they would of another person (i.e., “as object”), or whether they think of themselves “as subject.” In the case of exposure to emotional pictures, it seems plausible that subjects think of themselves “as subject.” The picture elicits a (more or less) intense emotional reaction, which the subject can then self-ascribe by means of introspection, that is, on the basis of their experience. As subjects only have privileged access to their own experiences in this way, this seems to be different from the way in which they would think of someone else. On the other hand, it might not always be apparent to me what emotional state I am in, and I might in some cases have to rely on inference in order to figure out what I am feeling – similar to when I am trying to figure out the emotional state of someone else. However, in the case of ascribing personality traits or bodily traits to oneself, the subjects seem to think of themselves “as object.” In order to be able to ascribe bodily traits to oneself, one needs to look at oneself, as one would at another (for instance, by looking at a mirror or a picture). Similarly, we often ascribe personality traits to ourselves on the basis of what other people (such as friends or family) tell us about ourselves, or by reflecting on past experiences and judging our own behavior in light of what it tells us about our personality. Again, this way of thinking about oneself is not in principle different from the way one would think about another.

What would be interesting is for future experiments to take into account this distinction in order to try to find out whether and how the distinction between thinking of oneself “as subject” and thinking of oneself “as object” is reflected in different patterns of neural activation. In order to do, paradigms would have to be developed to clearly distinguish between these two conditions. That is, the paradigms in question would have to be able to distinguish between judgments that are made based on ways of gaining information that are specific to the self (such as judgments made on the basis of the experience of a mental or bodily state) and those that are made based on ways of gaining information that are not specific to the self.

Although some studies have made first steps in that direction (Ochsner et al., 2005; Jenkins and Mitchell, 2011), they do not adequately distinguish between thinking of oneself “as subject” and thinking of oneself “as object.” For example, Ochsner et al. (2005) investigated the difference between direct and reflected self-appraisals, where the former was supposed to tap into the subject’s own beliefs about their traits, and the latter the subject’s perception of how others view them. They found that different judgment types all activated MPFC, though direct appraisals as compared to reflected appraisals also recruited the posterior cingulate, whereas reflected as compared to direct appraisals also recruited the insula, orbitofrontal, and temporal cortex. While these differences are interesting, note that the distinction between direct and reflected self-appraisals is not the same as the distinction between thinking of oneself “as subject” and thinking of oneself “as object.” This is because even in direct self-appraisals the subject needs to make herself an object of self-reflection in order to determine whether specific traits belong to herself. In contrast, when the subject thinks of herself “as subject,” she self-ascribes certain properties (such as the property of having a visual experience, feeling a pain or an emotion, or being an agent) in a direct, non-inferential way, on the basis of exploiting the self-specifying mechanisms that are implicit in conscious experience. In order to do so, she need not make herself an object of self-reflection; rather, she simply needs to apply the first-person concept in order to make explicit the self-specifying information that is implicit in her experience. (See below for further discussion of what this self-specifying information might consist in.)

Another recent study by Jenkins and Mitchell (2011) studied different types of self-reflection, namely reflection on one’s personality traits, reflection on current mental states, and reflection on bodily characteristics. They found that there was a robust region of MPFC that was more engaged when participants thought about themselves than when they made judgments about another person, regardless of the kind of self-reflection. They also found that differences in type of self-reflection were reflected in other areas of the brain, such as the TJP (for judgments of current mental states) or the cerebellum (for judgments of physical traits). But notice again that the different types of self-reflection studied here do not reflect the distinction between thinking of oneself “as subject” and thinking of oneself “as object.” This is because the different types of self-reflection under investigation here are distinguished in terms of their content. However, as I have argued above, the distinction between thinking of oneself “as subject” and thinking of oneself “as object” is not based on a difference in content, but rather on a difference between modes of presentation. So while reflection on one’s stable personality traits arguably requires thinking of oneself “as object,” thinking about one’s current mental states might either be based on direct experience of the latter (thinking of oneself “as subject”), or on inferences similar to those employed in reasoning about the mental states of others. After all, as I pointed out above, while we do have privileged access to some of our mental states (for instance, I do not normally need to rely on inference to know that I experience a pain), other mental states (such as a slight feeling of irritation) might only become apparent when I reflect on my recent behavior and infer that I must be feeling irritable. Indeed, given that the experimental paradigm is based on explicit self-reflection, it seems plausible that it will predominantly be the latter – thinking of oneself “as object” – that is being prompted here. Interestingly, Jenkins and Mitchell, 2011, p. 216) found that “reflecting on one’s own current mental states was specifically associated with activation in regions previously linked to inferences about the mental states of others, including medial parietal cortex and bilateral temporoparietal junction,” in line with the thought that such self-reflection relies on processes that are similar to reflecting on the mental states of others. This is in line with previous findings as well (e.g., Mitchell et al., 2005; Uddin et al., 2007; Lombardo et al., 2010)^2^. Similarly, while reflection on my bodily traits will usually be an instance of thinking of myself “as object,” I can also self-ascribe bodily properties (such as the property of having my legs crossed) on the basis of proprioceptive experience, and thus “as subject.” Thus, more research is needed to specifically address the question whether the philosophical distinction between thinking of oneself “as object” (in the third-person mode) and thinking of oneself “as subject” (in the first-person mode) is reflected in the neurobiology.

Moreover, it would also be interesting to enlarge the scope of the neurocognitive study of the self to include the more basic aspects of the subjectivity of conscious experience, of being a self or a subject of experience, rather than focusing on the ability to think about oneself alone (cf. Legrand, 2003, 2006, 2007a,b; Legrand and Ruby, 2009; Christoff et al., 2011). Again, while these are to be distinguished from explicit self-awareness (in the sense of thinking about or representing oneself), they provide the basis for the ability to think first-person thoughts “as subject” (i.e., first-person thoughts that are immune to error through misidentification). Moreover, while there is no reason to think that these basic aspects of being a self will also be related to CMS activity, as they arguably constitute a structural feature of all conscious experience and interaction with the world, they are nonetheless an important aspect of our understanding of the self^3^. As mentioned in the previous section, recently, Legrand and Ruby (2009) and Christoff et al. (2011) have suggested that these are instantiated by self-specifying processes, which implement what they call a functional self/non-self distinction in perception, action, cognition, and emotion^4^. After all, in order to be able to engage and interact with the environment, an organism must be able to (implicitly) distinguish between afferent signals arising as a result of the organism’s own efferent processes (i.e., reafference signals) and afferent signals arising as a result of environmental events (i.e., exafference signals) (see, e.g., Christoff et al., 2011, p. 105). The sense in which the self is specified by means of these processes is as a subject of perception, emotion, and cognition, as well as an agent – hence they can provide the basis for thinking of oneself “as subject,” that is, in the first-person mode.

Notice though that while Christoff et al. (2011), as well as Legrand and Ruby (2009) and Legrand (2006, 2007a) take these processes of implicit self-specification to result in a type of self-experience or self-awareness, as mentioned above, I would prefer to reserve this term for the ability to explicitly represent the self. While self-specifying processes are doubtlessly involved in processes of perception, action, cognition, and emotion at the subpersonal level, this does not imply that the self is represented in the content of experience at the personal level (and on my view the latter is required for genuine self-awareness; see Musholt, 2013 for detailed discussion). But setting aside this largely terminological issue, I agree with Legrand (2003, 2006, 2007a,b), Legrand and Ruby (2009), and Christoff et al. (2011) that insofar as both the study of the subjectivity of conscious experience and of the ability to represent oneself in thought are important avenues of research, one should not unnecessarily restrict the cognitive neuroscience of the self to the latter alone. Rather, one should attempt to study both of these aspects, albeit against a background of careful conceptual distinction.

### Implications for schizophrenia

Studying the implicitly self-specifying processes that are involved in perception, action, emotion, and cognition might also be interesting from a clinical perspective, as it could shed light on the neural correlates of disorders of the self, such as those occurring in patients suffering from schizophrenia. Recent research on schizophrenia suggests that both self-consciousness (in the sense of representing oneself) and the general structure of consciousness as such can be altered in patients suffering from this disorder.

Phenomenologically inclined authors have recently suggested that schizophrenia is an ipseity disturbance, or self-disorder, which manifests itself in both altered forms of self-awareness, as well as altered forms of consciousness more generally. Put differently, schizophrenia seems to affect both the “world-directed” and “self-directed” aspects of consciousness. As Sass and Parnas (2003) put it:

“[…] this ipseity disturbance has two fundamental and complementary aspects or components. The first is hyperreflexivity, which refers to forms of exaggerated self-consciousness in which a subject or agent experiences itself, or what would normally be inhabited as an aspect or feature of itself, as a kind of external object. The second is a diminishment of self-affection or auto-affection—that is, of the sense of basic self-presence, the implicit sense of existing as a vital and self-possessed subject of awareness.” (p. 428)

This is to say that patients suffering from schizophrenia seem to direct an unusual amount of attention toward aspects of their self that are normally not explicitly represented – the self becomes objectified, rather than being part of the subjective experience of engaging with the world. At the same time, the experience of oneself as a subject seems in some sense diminished or reduced. And further, these “complementary distortions are necessarily accompanied by certain kinds of alterations or disturbances of the subject’s ‘grip’ or ‘hold’ on the conceptual or perceptual field” (Sass and Parnas, 2003), suggesting that it is not just the conscious experience of oneself that is altered, but also more generally the conscious experience of the world. Put differently, the very structure of consciousness as such seems to be affected. The fact that ipseity disturbances in schizophrenia affect both how the world in general is experienced as well as leading to specific aspects of the self entering into the focus of attention is also stressed by Sass et al. (2011).

If this analysis of schizophrenia as an ipseity disturbance is right, and if, furthermore, Legrand and Ruby (2009) and Christoff et al. (2011) are right in stressing the role of implicitly self-specifying processes in various types of conscious experience, then it stands to reason that it is precisely these processes that are affected in schizophrenia. Accordingly, further study of these processes and their potential relation to ipseity disturbances in schizophrenia – in particular disturbances of the general structure of consciousness – could help to make progress in understanding the causes of schizophrenia. While some studies have already begun to address the relation between abnormal CMS function and abnormal self-reflection in schizophrenia (van der Meer et al., 2010), as well as with faulty interpretation of social events, which, in turn, might contribute to the development of delusions (Holt et al., 2011), it would be worthwhile to further investigate both the link between abnormal CMS function and abnormal self-reflection, as well as the link between abnormalities in implicitly self-specifying processes (independent of processes of self-reflection that are being linked to CMS) and ipseity disturbances.

Indeed, Christoff et al. (2011) mention two paradigmatic cases of such self-specifying processes, namely cognitive control and emotion regulation. They also point to various brain regions thought to be associated with these processes, in particular the lateral PFC, dorsomedial PFC, and dorsal ACC (for cognitive control and explicit emotional regulation), and the rostral ACC (rACC), subgenual ACC, and vmPFC (for implicit emotional regulation). It would be interesting to see whether these brain areas show a different pattern of activation in patients suffering from schizophrenia.

### The role of phenomenology

However, such an endeavor also requires careful conceptual and phenomenological analysis. As we have seen above, the study of the self is multi-faceted and one needs to be careful in detailing which aspect of the self or self-awareness one is intending to study and in showing that the paradigms employed do indeed capture the specific aspect or phenomenon in question. Phenomenology can help with such an analysis. Indeed, as various authors (e.g., Varela, 1996; Gallagher, 1997; Sass and Parnas, 2006; Thompson, 2007; Sass et al., 2011) have pointed out, the relation between phenomenology and cognitive neuroscience can be seen as being mutually constraining. This means that phenomenological analysis can provide part of the data set that neuroscientific theorizing needs to take into account. After all, a neuroscientific study of self and self-awareness cannot succeed without an understanding of what it is that one wants to explain, and phenomenology – as the study of the structure of awareness (including self-awareness) – can provide this understanding. At the same time, neuroscience can also provide data that challenge established ways of thinking about phenomenology, thus providing impulse for new ways of conceptualizing aspects of conscious experience. An example for this might be a case in which neuroscience highlights the involvement of distinct neurobiological systems in a type of experience that was previously analyzed as being unified. While this result need not necessarily lead to a change in the analysis of the conscious phenomenon in question (because one cannot simply assume that all aspects of subpersonal level processing are reflected at the personal level; see below), it can prompt further reflection that might ultimately reveal the phenomenon to be more complex than was previously thought (Sass et al., 2011).

To put the interaction between phenomenology and cognitive neuroscience into more concrete terms, one way (initially suggested by Varela, 1996) in which this interplay between phenomenology and neuroscience can be put into practice is by asking participants to describe their experiences by means of open-ended questionnaires, so as to avoid the imposition of pre-established theoretical categories. The resulting descriptions must then be validated intersubjectively in order to be usable for the interpretation of correlated measurements of brain activity and/or behavior (Sass et al., 2011, p. 5). At the same time, neuroscientific findings, for example the discovery of distinct systems underlying the experience of what appears to be a unified phenomenon, can motivate further investigation into the correct phenomenological analysis (Sass et al., 2011)^5^.

Another way of incorporating phenomenology into cognitive neuroscience is by relying on results from philosophical phenomenology (that is, on a philosophical analysis of the phenomenology of certain experiences, rather than on interviews with participants), and to employ these in order to inform the set-up of experiments. This is called “phenomenological front-loading” (Gallagher, 2003). Again, the methodology is not to be seen as a “one-way-street” – rather, there should be a dynamic interplay between phenomenological analysis and preliminary trials in the process of establishing the best experimental paradigm.

The emphasis on the need for such a two-way exchange between philosophy and neuroscience brings us to the third part of this paper, namely the relation between personal and subpersonal level explanations.

## The Personal and the Subpersonal in Neuroimaging the Self

At the personal level we refer to a person’s conscious experience and mental states, for example in order to make intelligible the behavior of a person by providing the reasons the person might have for acting in the way that they do. Subpersonal level explanations on the other hand provide information about the physiological or computational enabling conditions of personal-level phenomena. Properties at the subpersonal level are neither conscious, nor do they make reference to mental states or reasons.

Clearly, talk about the self and self-awareness is talk about personal-level phenomena. Likewise, phenomenological analyses are situated at the personal level – the level of the subject’s experience (or phenomenology). Brain imaging results, on the other hand, are situated at the subpersonal level.

It is important to keep these two levels separate in order to avoid the mistake of ascribing properties to one level that only belong to the other. For example, we can only properly ascribe conscious mental states to the person, not to parts of the person, such as the brain (or areas within the brain) (Bennett and Hacker, 2003). Likewise, as we have seen above, although there are implicitly self-specifying processes that form part of the cognitive mechanisms enabling the perception of and interaction with the environment, we cannot simply assume that the information contained in these processes is explicitly represented at the personal level. Rather, there are good reasons to think that in basic forms of perception and action the self is not explicitly represented (Musholt, 2013). Moreover, while the personal level is amenable to appeals to reason, the subpersonal level is not reasons-responsive.

If this is so, how should we think about the relation between personal and subpersonal level explanations?

There are three different ways of conceptualizing this relationship. First, personal-level phenomena can simply be correlated with subpersonal level phenomena without this implying either a causal or a constitutive relationship. On this view, neuroimaging studies can only provide us with information about the neural correlates of the self and self-awareness. Second, subpersonal level explanations can give us information about the causes or enabling conditions of personal-level phenomena. On this view, neuroscience can reveal the causes of certain personal-level phenomena, but it doesn’t tell us anything about what these phenomena really *are*. Third, subpersonal level explanations can enter into the constitutive conditions of personal-level phenomena. That is, they can enter into a conceptual understanding of what a specific phenomenon consists in.

Some philosophers (e.g., McDowell, 1994; Hornsby, 1997, 2000) have argued that the personal and the subpersonal are autonomous levels of explanation, and that information about subpersonal level processes can at best provide us with knowledge about the (causal) enabling mechanisms of personal-level phenomena, but it cannot tell us anything about the constitutive conditions of the latter. This is because personal-level explanations – explanations that proceed by reference to a person’s conscious mental states and their reasons for doing something – are of a distinct kind, so that it would be a mistake to try to combine such explanations with explanations of a very different kind, such as those that operate at the subpersonal level. While one type of explanation appeals to how things *ought* to be, by means of appealing to reasons, the other explains how things *happen* to be, by means of appeal to nomological generalizations (Hornsby, 1997; Shea, 2013).

Put differently, on this view, explanations that refer to reasons are constitutive of personal-level explanations. And since we cannot appeal to reasons at the subpersonal level, facts about what goes on at the subpersonal level cannot enter into explanations as to what constitutes personal-level phenomena (Colombo, 2013). As Hornsby puts it: “a study of the mechanisms of neural transmission won’t help in understanding what a person’s intentionally doing something *consists in*” (2000, p. 16; cited in Colombo, 2013, emphasis mine).

However, other philosophers (Colombo, 2013, Shea, 2013) have recently argued that even if the mental cannot be reduced to the neurobiological (and it seems plausible that it cannot), there might still be ways in which information about subpersonal level processes can contribute constitutive conditions for personal-level phenomena.

How so? Basically, according to Colombo (2013), such an interaction would be based on what Churchland (1986) has termed a co-evolutionary conception of the relationship between different explanatory levels. Such a co-evolution “ involves explanations and concepts at one level being susceptible to correction, reconceptualization, and sometimes elimination, in light of discoveries and conceptual refinements at other levels” (Colombo, 2013, p. 6).

This approach is exemplified by the aforementioned two-way-interaction between phenomenological and neuroscientific analysis, where neuroscientific results might in some case motivate the re-examination of phenomenological analysis, even if they cannot in and of themselves force a phenomenological redescription. If, based on neurobiological findings, we arrive at a different conceptualization of what a certain conscious experience *really is*, then, so it seems, this information will have made a contribution as to what *constitutes* the experience in question. After all, according to Hornsby and McDowell, a constitutive explanation provides us with an account of what a personal-level phenomenon, such as a person doing something intentionally *consists in*. Thus, an appeal to subpersonal mechanisms might – in certain circumstances – help us explain both the presence and break down of personal-level phenomena in ways that go beyond correlation (or even causation).

A concrete example for such an interaction that is discussed by Colombo (2013) involves the cases of addiction, pathological gambling, and “sex addiction.” At the personal level, both pathological gambling and “sex addiction” seem similar in the relevant respects; accordingly, one might classify them as instantiating the same phenomenon, namely a type of addiction. However, as Colombo points out, there is evidence suggesting that addiction has a particular neurocomputational signature. In particular, addiction seems to involve the engagement of the so-called “reward-system,” which relies on computations based on dopamine activity. In the case of addiction, an increased flow of dopamine into the reward-system leads to a loss of inhibition or impulse, thereby granting the system increased influence over the subject’s behavior. As a result,

“[…] the reward system simultaneously learns to pursue a target obsessively and increases the relative valuation of stimuli that predict it…. [It systematically pulls] attention back toward the addictive target, and away from the alternative motivators on which cortical systems are trying to focus.” (Ross, 2010, p. 138; cited in Colombo, 2013, p. 17)^6^

Now, as Colombo (2013) further points out, pathological gambling seems to share this neurocomputational signature. “Sex addiction,” on the other hand, doesn’t seem to involve the kind of changes to the dopaminergic reward-system observed in both addiction and pathological gambling (Ross et al., 2010). Moreover, the subpersonal facts about the neurocomputational mechanisms, in conjunction with what we know about gambling help us to understand while gambling can become addictive: it allows for perfect control over the cues that are predictive of reward (such a pressing a button on a slot machine), while providing no control over actual reward-contingencies (the actual winning of money). Neither of these features seems to be present when it comes to sex. Consequently, according to Colombo, while an understanding of the neurocomputational mechanisms of addiction helps us to understand pathological gambling *as* a form of addiction, the same cannot be said about “sex addiction.” Thus, it seems to be wrong to describe a certain type of behavior as a case of addiction to sex – knowledge about the mechanisms of addiction together with knowledge about the subpersonal processes that underlie gambling and “sex addiction” suggests that the label “sex addiction” constitutes a conceptual mistake. We can thus see that knowledge about subpersonal facts can form part of the constitutive (or conceptual) conditions for a personal-level phenomenon. This, in turn, can have important theoretical as well as practical implications, for instance in terms of developing treatments (Colombo, 2013).

A second example that is would like to consider, which is discussed by Shea (2013), refers us back to the importance of the cognitive neuroscience of the self and self-awareness. Consider again the implicit self-specifying mechanisms that are involved in perception, action, emotion, and cognition mentioned above. I have suggested above that a break down in these mechanisms might contribute to some of the symptoms associated with schizophrenia. In fact, it has recently been suggested that certain positive symptoms, such as auditory hallucinations or the loss of a sense of agency might be due to such a break down (Fletcher and Frith, 2009; Shea, 2013). In particular, the idea is that such symptoms can be traced back to changes in the way in which representations of the world are updated by error signals (which are, in turn, linked to abnormal dopamine neurotransmission). As we have seen above, self-specifying processes are generally explained in terms of a comparator model that distinguishes between afferent signals arising as a result of the organism’s own efferent processes (i.e., reafference signals) and afferent signals arising as a result of environmental events (i.e., exafference signals). Now, one hypothesis is that, say, in the case of auditory hallucinations, the mechanism that normally distinguishes between one’s own voice and that of another by means of such a comparison is impaired, such that the patient is no longer able to reliably tell the difference between the voices generated by others and their own inner speech. Normally, inner speech is predictable based on the subject’s belief and other mental states (in contrast to the speech of others). However, so the hypothesis, in the case of a break down of the normal comparator mechanism, the patient might receive a prediction-error signal when engaging in inner speech, which would lead them to conclude that the voice must have been generated by someone else (Shea, 2013).

Notice that this model is still controversial. However, we do not need to be concerned here with whether this is indeed an accurate model for the explanation of certain positive symptoms in schizophrenia. Rather, what matters for our present purposes is the point that if such a subpersonal level model could be established, it would go some way to explain what clearly is a personal-level phenomenon, such as the experience of auditory hallucinations. In particular, as Shea (2013) suggests, it might go some way in explaining why some of the features that are normally associated with the personal level, namely the rational connections between various mental states, break down. After all, according to this model, it is the very subpersonal mechanisms that normally enable an accurate representation of and rational response to evidence that are hypothesized to be affected, thereby explaining why such a rational response is no longer possible. Accordingly,

“[…] if a patient’s experience of hearing voices can be explained by a pathology in the subpersonal mechanisms that constitute the normal capacity for responding to evidence in rational ways, then we again have a subpersonal factor that is more than just an external cause of the personal level phenomenon. It is *part of the constitutive basis of that phenomenon*. But because it is operating abnormally, the nature of the personal level phenomenon itself changes.” (Shea, 2013, p. 17, emphasis added)

Thus, not only can knowledge about brain mechanisms be important in order to develop a causal understanding of (thereby contributing to the creation of better treatments of) pathological experiences and behaviors, but it can also contribute to the conceptual question as to what constitutes a specific phenomenon. This includes phenomena related to the self, such as the ability to distinguish between one’s own voice and those of others (or between one’s inner speech and the perception of the speech of others). Of course, this leaves open whether knowledge about cognitive processing in CMS does likewise have the potential to enter into the constitutive conditions for self-reflection.

It is important to be clear that localization studies as such remain at the level of correlating personal and subpersonal level phenomena. If we want to move beyond correlations and toward knowledge of causal, or even constitutive conditions, we need information about the specific mechanisms involved in bringing about personal-level phenomena. That is to say, we need functional (e.g., neurocomputational) analyses. Although they themselves cannot provide information about mechanisms, localization studies can provide an important first step in this direction because once we have identified the areas that are activated in certain cognitive processes, we can begin to develop hypotheses about their specific functional roles and the computational mechanisms that enable them to fulfill these roles.

## Conclusion

The leading question of this Research Topic was “Why and how is the self related to the brain midline regions?” As we have seen, while there is evidence to suggest that CMS do indeed play a privileged role in self-related processing, we have also seen that there is reason to doubt that the self is strictly speaking “located” in the CMS. This is because the CMS can also be shown to be involved in non-self-related cognitive tasks, while self-related processing also seems to involve brain areas outside of the CMS. Moreover, we have seen that it might not make much sense the speak of “the self” as such to begin with, as the notion of self and self-awareness is complex and multi-faceted.

Indeed, there are several philosophical distinctions that can be made with regard to the self and self-experience. This article focused on one of these, namely the distinction between being a self (i.e., being a subject of conscious experience) and being self-aware (i.e., being able to think about oneself), where the latter is closely tied to the ability to think about others. In addition, we saw that one can distinguish between thinking about oneself “as subject” (in the first-person mode) and “as object” (in the third-person mode). It was shown that studies of the relation between CMS and the self tend to focus on the ability to think about oneself, while neglecting the more basic aspects of being a self. Moreover, the sense of self-representation targeted in these studies seems to be mostly the sense of thinking of oneself “as object.” It was suggested that it might be worthwhile to target the distinction between thinking of oneself “as subject” and thinking of oneself “as object” in future studies to see whether this distinction is reflected in the neurobiology.

In addition, it was suggested that while the more basic aspects of being a self might not possess any particular relation to the CMS, it would nevertheless be wrong to restrict the cognitive neuroscience of the self to the ability to think about oneself, not least because a better understanding of the more basic aspects of being a self (i.e., certain aspects of conscious experience in general, such as the perspectivalness of conscious experience) might be important for a better understanding of disorders of the self, such as schizophrenia.

It was further argued that in the pursuit of the study of the self and self-awareness, cognitive neuroscience, and philosophy (in particular phenomenology) ought to work together.

Finally, it was shown with the help of two examples that while it is important to respect the distinction between the personal and the subpersonal, knowledge about subpersonal level processes can potentially contribute to a reconceptualization of personal-level phenomena, including those that are related to the self. However, this leaves open whether a particular pattern of activation in CMS can be considered part of the constitutive basis of being in a state of self-awareness. What the discussion about the relation between the personal and the subpersonal also shows is that in order for there to be a fruitful interaction between different levels of explanation, we need not only careful conceptual and phenomenological analysis, but also an understanding of cognitive mechanisms, which must go beyond facts about patterns of activation. That is to say that we need a functional analysis of subpersonal level processes. Localization studies can provide an important first step toward such a functional analysis, but they are not in and of themselves sufficient.

## Conflict of Interest Statement

The author declares that the research was conducted in the absence of any commercial or financial relationships that could be construed as a potential conflict of interest.

## Footnotes

^1^Though note that Schilbach et al. (2008) argue that activity in the default system of the brain is intimately linked to a human predisposition for social cognition. The relation between social cognition and self-awareness will be further discussed below.

^2^Though notice that since the ascription of mental and bodily states to oneself and to others (or to oneself “as object” and “as subject”) will always rely on a common conceptual schema (despite differences in mode of presentation), we should always expect some overlap between judgments involving the same concepts.

^3^Indeed, as Christoff et al. (2011) point out, it is likely that the widespread assumption that the self is “instantiated” by the default-network of the brain will be called into question if we expand the scope of the empirical study of the self. For it will be precisely in interactions with the external world that the more basic aspects of being a self will come into focus. Also see Legrand (2007a).

^4^The notion of self-specifying processes and their relevance for self-consciousness is also discussed at length by, for example, Bermúdez (1998) and Vosgerau (2009).

^5^For a detailed open-ended questionnaire along those lines, which has been developed on the basis of self-descriptions obtained from patients suffering from schizophrenia for the study of anomalous self-experience (see Parnas et al., 2005). For a discussion of how phenomenology could be incorporated into the study of auditory verbal hallucinations in particular (see McCarthy-Jones et al., 2013).

^6^But notice that the gambling need not be experienced as rewarding by the patient at the personal-level. In fact, it is precisely one of the hallmarks of addiction that patients themselves would like to stop the behavior in question, but find themselves being unable to do so, due to the subpersonal processes driving the behavior. So again, it is important to keep personal and subpersonal level explanations separate, even though there can be fruitful interaction between them.

## References

[B1] Baker L. R. (1998). The first-person perspective: a test for naturalism. Am. Philos. Q. 35, 327–348

[B2] Baker L. R. (2012). From consciousness to self-consciousness. Grazer Philos. Stud. 84, 19–38

[B3] Beckermann A. (2003). “Self-consciousness in cognitive systems,” in Persons: An Interdisciplinary Approach, eds Kanzian C. H. Quitterer J. Runggaldier E. (Wien: ÖBV & HPT), 174–188

[B4] Bennett M. R. Hacker P. M. S. (2003). Philosophical Foundations of Neuroscience. Oxford: Blackwell Publishing

[B5] Bermúdez J. L. (1998). The Paradox of Self-Consciousness. Cambridge, MA: MIT Press

[B6] Christoff K. Cosmelli D. Legrand D. Thompson E. (2011). Specifying the self for cognitive neuroscience. Trends Cogn. Sci. (Regul. Ed.) 15, 104–112 10.1016/j.tics.2011.01.001 21288760

[B7] Churchland P. S. (1986). Neurophilosophy: Toward a Unified Science of the Mind-Brain. Cambridge, MA: MIT Press

[B8] Colombo M. (2013). Constitutive relevance and the personal/subpersonal distinction. Philos. Psychol. 26, 547–570 10.1080/09515089.2012.667623

[B9] Evans G. (1982). The Varieties of Reference. Oxford: Oxford University Press

[B10] Fletcher P. C. Frith C. D. (2009). Perceiving is believing: a Bayesian approach to explaining the positive symptoms of schizophrenia. Nat. Rev. Neurosci. 10, 48–58 10.1038/nrn2536 19050712

[B11] Gallagher S. (1997). Mutual enlightenment: recent phenomenology in cognitive science. J. Conscious. Stud. 4, 195–214

[B12] Gallagher S. (2003). Phenomenology and experimental design toward a phenomenologically enlightened experimental science. J. Conscious. Stud. 10, 9–10

[B13] Gillihan S. J. Farah M. J. (2005). Is self special? A critical review of evidence from experimental psychology and cognitive neuroscience. Psychol. Bull. 131, 76–97 10.1037/0033-2909.131.1.76 15631554

[B14] Gusnard D. A. Akbudak E. Shulman G. L. Raichle M. E. (2001). Medial prefrontal cortex and self-referential mental activity: relation to a default mode of brain function. Proc. Natl. Acad. Sci. U.S.A. 98, 4259–4264 10.1073/pnas.071043098 11259662 PMC31213

[B15] Heinzel A. Walter M. Schneider F. Rotte M. Matthiae C. Tempelmann C. (2006). Self-related processing in the sexual domain: a parametric event-related fMRI study reveals neural activity in ventral cortical midline structures. Soc. Neurosci. 1, 41–51 10.1080/17470910600663137 18633774

[B16] Holt D. J. Lakshmanan B. Freudenreich O. Goff D. C. Rauch S. L. Kuperberg G. R. (2011). Dysfunction of a cortical midline network during emotional appraisals in schizophrenia. Schizophr. Bull. 37, 164–176 10.1093/schbul/sbp067 19605517 PMC3004194

[B17] Hornsby J. (1997). Simple Mindedness: In Defense of Naive Naturalism in the Philosophy of Mind. Cambridge, MA: Harvard University Press

[B18] Hornsby J. (2000). Personal and sub-personal: a defence of Dennett’s early distinction. Philos. Explor. 3, 6–24 10.1080/13869790008520978

[B19] Jenkins A. C. Mitchell J. P. (2011). Medial prefrontal cortex subserves diverse forms of self-reflection. Soc. Neurosci. 6, 211–218 10.1080/17470919.2010.507948 20711940

[B20] Keenan J. P. Nelson A. O’Connor M. Pascual-Leone A. (2001). Self-recognition and the right hemisphere. Nature 409, 305 10.1038/35053167 11201730

[B21] Kelley W. M. Macrae C. N. Wyland C. L. Caglar S. Inati S. Heatherton T. F. (2002). Finding the self? An event-related fMRI study. J. Cogn. Neurosci. 14, 785–794 10.1162/08989290260138672 12167262

[B22] Legrand D. (2003). How not to find the neural signature of self-consciousness (commentary on Newen & Vogeley). Conscious. Cogn. 12, 544–546 14656496 10.1016/s1053-8100(03)00080-1

[B23] Legrand D. (2006). The bodily self: the sensori-motor roots of pre-reflexive self-consciousness. Phenomenol. Cogn. Sci. 5, 89–118 10.1007/s11097-005-9015-6

[B24] Legrand D. (2007a). Pre-reflective self-as-subject from experiential and empirical perspectives. Conscious. Cogn. 16, 583–599 10.1016/j.concog.2007.04.002 17533140

[B25] Legrand D. (2007b). Subjectivity and the body: introducing basic forms of self-consciousness. Conscious. Cogn. 16, 577–582 10.1016/j.concog.2007.06.011 17716918

[B26] Legrand D. Ruby P. (2009). What is self-specific? Theoretical investigation and critical review of neuroimaging results. Psychol. Rev. 116, 252–282 10.1037/a0014172 19159156

[B27] Lombardo M. V. Chakrabarti B. Bullmore E. T. Wheelwright S. J. Sadek S. A. Suckling J. (2010). Shared neural circuits for mentalizing about the self and others. J. Cogn. Neurosci. 22, 1623–1635 10.1162/jocn.2009.21287 19580380

[B28] McCarthy-Jones S. Krueger J. Larøi F. Broome M. Fernyhough C. (2013). Stop, look, listen: the need for philosophical perspectives on auditory verbal hallucinations. Front. Hum. Neurosci. 7:127 10.3389/fnhum.2013.00127 23576974 PMC3620561

[B29] McDowell J. (1994). Mind and World. Cambridge, MA: Harvard University Press

[B30] Mitchell J. P. Banaji M. R. Macrae C. N. (2005). The link between social cognition and self-referential thought in the medial prefrontal cortex. J. Cogn. Neurosci. 17, 1306–1315 10.1162/0898929055002418 16197685

[B31] Modinos G. Ormel J. Aleman A. (2009). Activation of anterior insula during self-reflection. PLoS ONE 4:e4618 10.1371/journal.pone.0004618 19242539 PMC2643476

[B32] Moran J. M. Heatherton T. F. Kelley W. M. (2009). Modulation of cortical midline structures by implicit and explicit self-relevance evaluation. Soc. Neurosci. 4, 197–211 10.1080/17470910802250519 19424905 PMC4532268

[B33] Musholt K. (2012). Self-consciousness and intersubjectivity. Grazer Philos. Stud. 84, 63–89

[B34] Musholt K. (2013). Self-consciousness and nonconceptual content. Philos. Stud. 163, 649–672 10.1007/s11098-011-9837-8

[B35] Northoff G. Bermpohl F. (2004). Cortical midline structures and the self. Trends Cogn. Sci. (Regul. Ed.) 8, 102–107 10.1016/j.tics.2004.01.004 15301749

[B36] Northoff G. Heinzel A. de Greck M. Bermpohl F. Dobrowolny H. Panksepp J. (2006). Self-referential processing in our brain? A meta-analysis of imaging studies on the self. Neuroimage 31, 440–457 10.1016/j.neuroimage.2005.12.002 16466680

[B37] Northoff G. Schneider F. Rotte M. Matthiae C. Tempelmann C. Wiebking C. (2009). Differential parametric modulation of self-relatedness and emotions in different brain regions. Hum. Brain Mapp. 30, 369–382 10.1002/hbm.20510 18064583 PMC6870760

[B38] Ochsner K. N. Beer J. S. Robertson E. R. Cooper J. C. Gabrieli J. D. Kihsltrom J. F. (2005). The neural correlates of direct and reflected self-knowledge. Neuroimage 28, 797–814 10.1016/j.neuroimage.2005.06.069 16290016

[B39] Parnas J. Møller P. Kircher T. Thalbitzer J. Jansson L. Handest P. (2005). EASE: examination of anomalous self-experience. Psychopathology 38, 236–258 10.1159/000088441 16179811

[B40] Perry J. (1998). “Indexicals, contexts and unarticulated constituents,” in Proceedings of the 1995 CSLI-Amsterdam Logic, Language and Computation Conference (Stanford: CSLI Publications), 1–16

[B41] Perry J. (2002). “The self, self-knowledge, and self-notions,” in Identity, Personal Identity, and the Self, ed. Perry J. (Indianapolis: Hackett Publishing), 280

[B42] Platek S. M. Keenan J. P. Gallup G. G. Jr. Mohamed F. B. (2004). Where am I? The neurological correlates of self and other. Cogn. Brain Res. 19, 114–122 10.1016/j.cogbrainres.2003.11.014 15019708

[B43] Platek S. M. Wathne K. Tierney N. G. Thomson J. W. (2008). Neural correlates of self-face recognition: an effect-location meta-analysis. Brain Res. 1232, 173 10.1016/j.brainres.2008.07.010 18656465

[B44] Qin P. Northoff G. (2011). How is our self related to midline regions and the default-mode network? Neuroimage 57, 1221–1233 10.1016/j.neuroimage.2011.05.028 21609772

[B45] Recanati F. (2012). “Immunity to error through misidentification: what it is and where it comes from,” in Immunity to Error through Misidentification: New Essays, eds Prosser S. Recanati F. (Cambridge: Cambridge University Press), 180–201

[B46] Ross D. (2010). “Economic models of pathological gambling,” in What is Addiction? eds Ross D. Kincaid D. Spurrett D. Collins P. (Cambridge, MA: MIT Press), 131–158

[B47] Ross D. Sharp C. Vuchinich R. Spurrett D. (2010). Midbrain Mutiny: The Picoeconomics and Neuroeconomics of Disordered Gambling. Cambridge, MA: MIT Press

[B48] Sass L. Parnas J. Zahavi D. (2011). Phenomenological psychopathology and schizophrenia: contemporary approaches and misunderstandings. Philos. Psychiatr. Psychol. 18, 1–2318924253

[B49] Sass L. A. Parnas J. (2003). Schizophrenia, consciousness, and the self. Schizophr. Bull. 29, 427–444 10.1093/oxfordjournals.schbul.a007017 14609238

[B50] Sass L. A. Parnas J. (2006). “Explaining schizophrenia: the relevance of phenomenology,” in Reconceiving Schizophrenia, eds Chung M. Fulford W. Graham G. (Oxford: Oxford University Press), 63–96 10.1093/med/9780198526131.003.0004

[B51] Schilbach L. Eickhoff S. B. Rotarska-Jagiela A. Fink G. R. Vogeley K. (2008). Minds at rest? Social cognition as the default mode of cognizing and its putative relationship to the “default system” of the brain. Conscious. Cogn. 17, 457–467 10.1016/j.concog.2008.03.013 18434197

[B52] Schneider F. Bermpohl F. Heinzel A. Rotte M. Walter M. Tempelmann C. (2008). The resting brain and our self: self-relatedness modulates resting state neural activity in cortical midline structures. Neuroscience 157, 120–131 10.1016/j.neuroscience.2008.08.014 18793699

[B53] Shea N. (2013). “Neural mechanisms of decision-making and the personal level,” in The Oxford Handbook of Philosophy and Psychiatry, eds Fulford K. W. M. Davies M. Gipps R. Graham G. Sadler J. Stanghellini G. Thornton T. (Oxford: Oxford University Press), 1063–1082

[B54] Shoemaker S. (1968). Self-reference and self-awareness. J. Philos. 65, 555–567 10.2307/2024121

[B55] Thompson E. (2007). Mind in Life: Biology, Phenomenology, and the Sciences of Mind. Cambridge: Harvard University Press

[B56] Uddin L. Q. Iacoboni M. Lange C. Keenan J. P. (2007). The self and social cognition: the role of cortical midline structures and mirror neurons. Trends Cogn. Sci. (Regul. Ed.) 11, 153–157 10.1016/j.tics.2007.01.001 17300981

[B57] van der Meer L. Costafreda S. Aleman A. David A. S. (2010). Self-reflection and the brain: a theoretical review and meta-analysis of neuroimaging studies with implications for schizophrenia. Neurosci. Biobehav. Rev. 34, 935–946 10.1016/j.neubiorev.2009.12.004 20015455

[B58] Varela F. J. (1996). Neurophenomenology: a methodological remedy for the hard problem. J. Conscious. Stud. 3, 330–349

[B59] Vosgerau G. (2009). Mental Representation and Self-consciousness: From Basic Self-Representation to Self-Related Cognition Paderborn: Mentis

[B60] Wicker B. Ruby P. Royet J. P. Fonlupt P. (2003). A relation between rest and self in the brain? Brain Res. Rev. 43, 224–230 10.1016/j.brainresrev.2003.08.003 14572916

[B61] Wittgenstein L. (1958). The Blue and Brown Books. Oxford: Blackwell

[B62] Yaoi K. Osaka N. Osaka M. (2009). Is the self special in the dorsomedial prefrontal cortex? An fMRI study. Soc. Neurosci. 4, 455–463 10.1080/17470910903027808 19588282

[B63] Zahavi D. (2005). Subjectivity and Selfhood: Investigating the First-Person Perspective. Cambridge, MA: MIT Press

